# Digital Interventions for Improving Positive and Negative Symptoms in Psychosis: A Systematic Review of Evidence.

**DOI:** 10.1192/bjo.2025.10195

**Published:** 2025-06-20

**Authors:** Nnamdi Anyim, Ahmed Waqas

**Affiliations:** ^1^Greater Manchester Mental Health (GMMH) Trust, Manchester, United Kingdom; ^2^University of Manchester, Manchester, United Kingdom

## Abstract

**Aims:** Digital interventions have gained prominence as effective tools for improving outcomes across various psychiatric disorders. These interventions offer scalability, thus enhancing access to mental health services, particularly in resource-limited settings. While substantial evidence supports the efficacy of digital interventions for neurotic disorders, there is a notable gap in data regarding their effectiveness in severe, enduring mental illnesses like schizophrenia. This systematic review aims to evaluate the types of digital interventions used in the treatment of psychotic disorders and assess their effectiveness in improving both positive and negative symptoms.

**Methods:** We conducted a comprehensive search of multiple databases, including PubMed, up to December 2024, for studies evaluating digital interventions in psychotic disorders using a pre-tested search strategy. After retrieving relevant bibliographic records, two reviewers independently screened titles, abstracts, and full texts. Data extraction was performed by both reviewers working separately. Key variables extracted included study design, intervention type and platform, target population, and intervention components. The primary outcomes assessed were the severity of positive and negative symptoms following the intervention.

**Results:** Nine studies met the inclusion criteria. Three studies focused on interventions for participants with schizophrenia (n=3), two studies focused on first episode psychosis (n=2), and four studies were on schizophrenia spectrum disorders (n=4). Of these, four studies employed randomized controlled trial (RCT) designs, while the remaining studies assessed intervention development (n=1) and feasibility (n=4). The digital platforms used in the interventions included apps designed for symptom monitoring, self-management, and early relapse identification. Notable features of the apps included affect monitoring (n=3), diary keeping (n=2), reminders (n=3), and peer networking (n=3). Two apps specifically aimed at monitoring early relapse used active symptom tracking (n=1) and passive behavioural monitoring via GPS (n=1). The intervention strategies were rooted in psychosocial principles, with some apps utilizing cognitive-behavioural therapy for psychosis (CBTp; n=4) and behavioural activation for exercise engagement (n=1). One study combined the Actissist and CliniTouch apps, with the CliniTouch app prompting users to respond to PANSS-based items, while the Actissist app delivered CBTp-based interventions. Positive outcomes were observed in areas such as paranoia, delusions, motivation, and overall PANSS scores.

**Conclusion:** The use of digital interventions in the treatment of psychotic disorders has increased, with diverse strategies showing potential for improving both mental and physical health outcomes. Although promising, further randomized controlled trials are necessary to establish firm recommendations for integrating these digital tools into clinical practice.

